# Prevalence of Perinatal Asphyxia in Neonates at a Tertiary Care Hospital: A Descriptive Cross-sectional Study

**DOI:** 10.31729/jnma.4550

**Published:** 2019-10-31

**Authors:** Sunil Raja Manandhar, Rydam Basnet

**Affiliations:** 1 Department of Pediatrics, Kathmandu Medical College Teaching Hospital, Sinamangal, Kathmandu, Nepal

**Keywords:** *apgar score*, *hypoxic ischemic encephalopathy*, *perinatal asphyxia.*

## Abstract

**Introduction:**

Perinatal asphyxia is one of the major causes of perinatal and early neonatal mortality in developing countries. The main objective of this study was to observe the prevalence of perinatal asphyxia in babies born at Kathmandu Medical College Teaching Hospital.

**Methods:**

This was a descriptive cross-sectional study conducted at Kathmandu Medical College Teaching Hospital over six month period (January to June 2019). All preterm, term and post term babies delivered at Kathmandu Medical College Teaching Hospital were included. Ethical clearance was received from Institutional Review Committee of Kathmandu Medical College (Ref.:2812201808). Convenient sampling method was applied. Data analysis was done in Statistical Package for Social Sciences version 18, point estimate at 95% Confidence Interval was calculated along with frequency and proportion for binary data.

**Results:**

A total of 1284 babies delivered over six months period were enrolled in this study and 47 (3.66%) babies were asphyxiated, at 95% Confidence Interval (2.64-4.68%). The mean birth weight of asphyxiated babies was 2759.75±65 grams and gestational age was 37.57±2 weeks. Among asphyxiated babies, 15 (32%) babies were normal, 15 (32%) babies were in Hypoxic Ischemic Encephalopathy stage I, 14 (30%) were in stage II and 3 (6%) were in stage III. Twenty Three (49%) asphyxiated babies had antenatal risk factors and all 47 babies had intrapartum risk factors leading to asphyxia.

**Conclusions:**

Prevalence of perinatal asphyxia was lower compared to that of other similar tertiary care hospitals. Perinatal asphyxia remains a major cause of neonatal morbidity and mortality.

## INTRODUCTION

Perinatal asphyxia is one of the major causes of perinatal and early neonatal mortality in developing countries contributing one quarter of the world’s three million neonatal deaths and almost half of 2.6 million third trimester stillbirths.^1,2^ Every year approximately 4 million babies are born asphyxiated resulting 1 million deaths and 1 million serious neurological consequences ranging from cerebral palsy and mental retardation to epilepsy.^2^

The major complication of perinatal asphyxia is Hypoxic Ischemic Encephalopathy (HIE). HIE is a syndrome of disturbed neurological function manifested by difficulty in initiating and maintaining respiration, abnormal muscle tone and reflexes, subnormal level of consciousness and often seizures.^3^ It is classified in different stages by Levene Classification.^4^ In severe HIE (HIE stage III), the newborn is comatose, severely hypotonic, prolonged seizure and is unable to sustain spontaneous respiration.^5^

The main objective of this study was to observe the prevalence of perinatal asphyxia in babies born at Kathmandu Medical College Teaching Hospital.

## METHODS

A descriptive cross-sectional study was carried out on neonates with perinatal asphyxia at 10 bedded Neonatal Intensive Care Unit (NICU) of Pediatrics Department, Kathmandu Medical College Teaching Hospital (KMCTH), Sinamangal, Kathmandu, Nepal over six months period (Jan 2019 to June 2019). Ethical clearance was received from Institutional Review Committee (IRC) of Kathmandu Medical College (Ref.:2812201808) and written consent was taken from the parents and possible complications of perinatal asphyxia were explained. Data were entered in Excel and analysis was done in SPSS 18, point estimate at 95% Confidence Interval was calculated along with frequency and proportion for binary data.

Perinatal Mortality Rate (PMR) of this tertiary hospital is 10/1000 births and Neonatal mortality rate (NMR) is 4.5/1000 live births.^6^ All preterm, term and post term babies born at KMCTH with perinatal asphyxia due to any antepartum cause (e.g. Pregnancy Induced Hypertension (PIH), pre-eclempsia/eclempsia, Oligohydramnios etc.) or Intrapartum cause (e.g. Meconium stain liquor, prolonged labor, prolonged 2^nd^ stage of labor, Obstructed labor) were included in this study. Babies with lethal congenital anomalies (eg. Meningomyelocele, Anencephaly, Gastroschisis, Diaphragmatic Hernia), Syndromic babies and out born neonates with perinatal asphyxia were excluded.

Neonates were diagnosed as perinatal asphyxia when at least three of the following four criteria were fulfilled:^7^

1. Umbilical Arterial cord pH <7.2 determined by blood gas analysis within first hour of birth
2. Apgar score ≤6 at 5 minute
3. Requirement of >1 minute of positive pressure ventilation
4. Signs of fetal distress (heart rate of less than 100 beats per minute, late decelerations, or an absence of heart rate variability)

Detailed obstetric history, birth history, risk factors in the pregnancy, type of delivery and need of resuscitation procedure required at birth were recorded. During resuscitation in labor room/operation theatre, Neonatal Resuscitation Program (NRP) guideline 2015 recommended by American Academy of Pediatrics (AAP) were followed and detailed neurological examination of asphyxiated newborns were performed.^8^ The staging of HIE was assessed by Leven’s classification.^5^ All neonates were managed according to hospital protocol.

sample size estimation

$$\text{n}=\frac{{\text{Z}}^{\text{2}}\times \text{p}\times \text{q}}{{\text{e}}^{\text{2}}}$$

where,

n = Sample size
Z = 1.96 for Confidence Interval of 95%
p = Prevalence of perinatal asphyxia in previous study = 15.9%^9^
q = 1-p
e = margin of error= 2%

$$\begin{array}{l} \text{n}=\frac{{\left(1.96\right)}^{2}\times 0.159\times \left(1-0.159\right)}{{\left(0.02\right)}^{2}}\\ =\frac{3.84\times 0.159\times 0.841}{0.0004}=\frac{0.5134}{0.0004}=1283.5\\ \text{n}=\text{1284}\;\text{neonates} \end{array}$$

Total of 1284 neonates were enrolled and convenient sampling method was applied. Statistical analysis was done with SPSS 18 version. Outcome and possible morbid conditions and sequelae due to HIE were observed.

## RESULTS

A total of 1284 babies over six month’s period were enrolled in this study. Out of which 47 babies were asphyxiated with a prevalence of perinatal asphyxia at KMCTH is 3.66%, at 95% Confidence Interval (2.64%-4.68%). Among 47 asphyxiated babies, 35 (75%) were term babies with male predominance 33 (70%). The mean birth weight of asphyxiated babies was 2759.57±65gms and mean gestational age was 37.57±2 weeks. Similarly, mean Apgar score at 1 min was 3.28±0.7 and at 5 min was 5.15±0.8. Mean maternal age of asphyxiated babies was 25.5±3.2yrs and mean maternal BMI was 20.04±2.4 kg/m^2^. Mean fetal heart sound (FHS) during admission at hospital was 132.60±18.1/min whereas during the delivery of baby, FHS has dropped to 88.87± 6.3/min depicted (Table 1).

**Table 1:** Demographics and maternal characteristics.

Variables
A.	Neonatal Characteristics	Mean	Range
1	Gestational Age	37.57±2	(32–40) wks.
2	Birth weight	2759.57±65	(1500-4000) gms
3	Apgar Score at 1 min (47 babies)	3.28±0.7	(1–5)
4	Apgar Score at 5 min (47 babies)	5.15±0.8	(3-6)
5	Apgar Score at 10 min (47 babies)	7.49±0.9	(5-9)
6	Apgar Score at 15 min (15 babies)	8.73±0.9	(7-10)
10	Average Hospital Stay (45 babies)	7±3.4	(3-15) days
B.	Maternal Characteristics	Mean	Range
1	Mothers Age	25.5±3.2	(18–38) yrs.
2	Mother’s BMI	20.04±2.4	(18-31) kg/m^2^
3	Maternal PROM (8 mothers)	30±6.2	(20-40) hrs
4	Maternal FHS during Admission	132.60±18.1	(60-165)/min
5	Maternal FHS at the time of delivery	88.87±6.3	(70-98) min

Twenty Three (49%) asphyxiated babies had antenatal risk factors and all 47 (100%) babies had intrapartum risk factors leading to asphyxia. Intra uterine growth factor (IUGR) among 11 (24%) was the commonest antenatal risk factor whereas prolonged labor 15 (32%) was the commonest intrapartum risk factor. Ten (21%) asphyxiated babies were cried by tactile stimulation only whereas 24 (51%) asphyxiated babies required Bag and Mask ventilation, 4 (9%) required Bag and mask with Chest compression and 6 (13%) babies were intubated. Only 3 (6%) severely asphyxiated babies required all form of resuscitation measures as per NRP guidelines 2015. Out of 47 asphyxiated babies, 32 (68%) developed HIE. Out of which 15 (32%) were in HIE stage I, 14 (30%) were in HIE stage II and 3 (6%) babies were in HIE stage III. All three babies with HIE stage III were intubated and required all form of resuscitation procedure including drugs at birth. (Table 2).

**Table 2:** Neonatal characteristics and clinical profile.

S.N.	Determinant	Characteristics	n (%)
1	Gender	Male	33 (70)
		Female	14 (30)
		Total	47 (100)
2	Gestation	Preterm	12 (25)
		Term	35 (75)
		Total	47 (100)
3	Gestational Age	SGA	10 (21)
		AGA	33 (70)
		LGA	4 (9)
		Total	47 (100)
4	Mode of delivery	LSCS	27 (58)
		ND	11 (23)
		Vacuum	7 (15)
		Forceps	2 (4)
		Total	47 (100)
5	Delivery conducted by	Doctor	46 (98)
		Nurse	1 (2)
		Total	47 (100)
6	Antenatal risk Factor	No risk factor	24 (51)
		IUGR	11 (24)
		Gestational Diabetes	8 (17)
		Maternal PROM	2 (4)
		Severe Pre-eclempsia	1 (2)
		Placenta previa	1 (2)
		Total	47 (100)
7	Intrapartum risk factor	Prolonged labor	15 (32)
		Thick meconium stain liquor	13 (28)
		Fetal Bradycardia	8 (17)
		Obstructed labor	7 (15)
		Precipitate labor	2 (4)
		Umbilical Cord prolapse	2 (4)
		Total	47 (100)
8	Resuscitation procedure required	Bag and Mask Ventilation	24 (51)
		Tactile stimulation	10 (21)
		Intubation	6 (13)
		Bag n Mask with Chest compression	4 (9)
		All procedures	3 (6)
		Total	47 (100)
9	HIE (Hypoxic ischemic encephalopathy) staging	No HIE	15 (32)
		HIE stage I	15 (32)
		HIE stage II	14 (30)
		HIE stage III	3 (6)
		Total	47 (100)

Out of 32 neonates with HIE, 24 (75%) babies’ 1 min Apgar score was 0-3 and 30 (94%) babies’ 5 min Apgar score was 4-6. Similarly, 19 (59%) babies with HIE had antenatal risk factors and all 32 (100%) babies with HIE had intrapartum risk factors. Among 32 HIE babies, 19 (59%) required Bag and Mask ventilation, 4 (13%) required Bag and mask with chest compression, 6 (19%) required Intubation and 3 (9%) required all procedures of neonatal resuscitation at birth (Table 3).

Outcome of total 47 asphyxiated babies were observed as follows: 45 (95.7%) babies were discharged and 2 (4.3%) babies with HIE stage III were expired. After following 45 asphyxiated babies till one month of postnatal age, 44 (93.6%) babies were neurologically normal and one baby (2.1%) survived with HIE stage III has developed spastic cerebral palsy (Table 4).

**Table 3:** Factors responsible for development of HIE.

S.N.	Determinant	Characteristics	HIE n (%)	No HIE n (%)
1	Gender	Male	24 (75)	9 (60)
		Female	8 (25)	6 (40)
		Total	32 (100)	15 (100)
2	Gestational	SGA	6 (19)	4 (27)
		AGA	23 (72)	10 (67)
		LGA	3 (9)	1 (6)
		Total	32 (100)	15 (100)
3	1 min Apgar Score	0-3	24 (75)	6 (40)
		4-6	8 (25)	9 (60)
		Total	32 (100)	15 (100)
4	5 min Apgar score	0-3	2 (6)	-
		4-6	30 (94)	15 (100)
		Total	32 (100)	15 (100)
5	Antenatal risk factor	Absent	13 (41)	11 (73)
		Present	19 (59)	4 (27)
		Total	32 (100)	15 (100)
7	Intrapartum risk factor	Absent	-	4 (27)
		Present	32 (100)	11 (73)
		Total	32 (100)	15 (100)
8	Resuscitation procedure required	Tactile stimulation only	-	10 (67)
		Bag and Mask	19 (59)	5 (33)
		Bag and Mask with Chest Compression	4 (13)	-
		Intubation	6 (19)	-
		All	3 (9)	-
		Total	32 (100)	15 (100)

**Table 4:** Outcome of perinatal asphyxia and its relationship with the hypoxemic ischemic encephalopathy staging.

S.N.	Variables	Characteristics	No HIE	HIE I	HIE II	HIE III	Total n (%)
1	Outcome of HIE	Discharged without sequelae	15	15	14	1	44 (93.6)
		Discharged with sequelae	-	-	-	1	1 (2.1)
		Expired	-	-	-	2	2 (4.3)
		Total	15	15	14	3	47 (100)
2	Hospital stay	1-7 days	15	11	4	-	30 (66.7)
		8-14 days	-	4	9	-	13 (28.9)
		>14 days			1	1	2 (4.4)
		Total	15	15	14	1	45 (100)

## DISCUSSION

Perinatal asphyxia contributes significantly to neonatal morbidity and mortality.^10,11^ In our study, the prevalence of perinatal asphyxia is 3.66%. This finding is quite low as compared to other studies done in Nepal as in Nepal Medical College 15.9% by Shrestha S et al,^9^ 26.9% at Kathmandu University School of Medical Sciences, Dhulikhel by Dangol S et al,^12^ 17.3% at College of Medical Sciences, Bharatpur by Gupta SK et al^13^ and 19.3% at Lumbini Medical College Teaching Hospital by Panthee K et al.^14^ The possible factors for such a low prevalence of perinatal asphyxia in KMCTH could be due to better intrapartum fetal monitoring (using Doppler Fetoscope, availability of USG in labor room, proper use of partographs during labor period) and immediate interventions like emergency cesarean section and instrumental delivery which have helped in reducing intrauterine asphyxia. Availability of level III neonatal care like bubble CPAP, mechanical ventilator support and surfactant replacement therapy at NICU and floor night duty of second on call Obstetricians and Pediatricians with Post Graduate resident doctors involved in every high risk deliveries might have helped in reducing perinatal asphyxia and immediate better neonatal resuscitation care could have reduced its complications of perinatal asphyxia.

In our study, majority of the asphyxiated babies 33 (70%) were appropriate of gestational age (AGA). This is similar to Kempegowda study by Sarnappa SB et al. where 80% were AGA.^7^ In our study, 5 min Apgar score 4-6, intrapartum risk factors and need of intubation and chest compression during neonatal resuscitation at birth were the major contributing factors leading to HIE. Whereas in a study done by Joseph S et al. in Kerala found need for Endotracheal intubation, Instrumental delivery and umbilical arterial pH <7 were the leading factors associated with HIE.^15^

In our study, 32 (68%) babies with perinatal asphyxia developed HIE, out of which only three babies (6%) were in HIE stage III. Out of these three babies, two babies (67%) expired and one baby (33%) developed neurological sequelae. Whereas a study done by Aliyu et al in Nigeria found 96% asphyxiated babies developed HIE and 34 (13.8%) were in HIE stage III.^16^ Out of those 34 HIE stage III babies, 4 (11.7%) babies developed neurological sequelae and 9 (26.4%) babies expired highlighting that HIE stage III babies were very difficult to survive and if survived, most of them will have sequelae.

## CONCLUSIONS

Perinatal asphyxia still remains major cause of neonatal mortality and morbidities particularly in developing countries. This study highlighted, even with low prevalence of perinatal asphyxia, intrapartum risk factors eg. thick meconium stain liquor, prolonged labor and requirements of neonatal resuscitation procedure eg. Intubation and Bag and mask with chest compression were the common risk factors associated with HIE. Since, this is a single institutional study with convenient sampling, outcome cannot be generalized.
